# A clinical study incorporating multimodal ^18^F-FDG PET/CT metabolic parameters, genetic markers, and clinical characteristics for the evaluation and prediction of treatment efficacy and prognosis in Langerhans cell histiocytosis

**DOI:** 10.3389/fmed.2025.1619967

**Published:** 2025-09-03

**Authors:** Zizhen Huang, Wanling Qi, Tian Rao, Qingyun Zeng, Ping Lu, Jie Zhang, Zhibin Qiu, Guihua Xiao, Qian Liu, Huan Fu, Fengxiang Liao

**Affiliations:** ^1^Department of Psychosomatic Medicine, Jiangxi Provincal People's Hospital (The First Affiliated Hospital of Nanchang Medical College), Nanchang, China; ^2^Department of Nuclear Medicine, Jiangxi Provincial People's Hospital (The First Affiliated Hospital of Nanchang Medical College), Nanchang, China; ^3^Nanchang YiMai Sunshine Medical Imaging Diagnosis Co., Ltd., Nanchang, China; ^4^Department of Cardiology, Jiangxi Provincial People's Hospital (The First Affiliated Hospital of Nanchang Medical College), Nanchang, China; ^5^Department of Pathology, Jiangxi Provincial People's Hospital (The First Affiliated Hospital of Nanchang Medical College), Nanchang, China; ^6^Department of Hematology, Jiangxi Provincial People's Hospital (The First Affiliated Hospital of Nanchang Medical College), Nanchang, China

**Keywords:** metabolic parameters, genetic markers, treatment efficacy, prognosis, Langerhans cell histiocytosis

## Abstract

**Objectives:**

Langerhans cell histiocytosis (LCH) is a rare clonal proliferative disorder characterized by the infiltration of pathological Langerhans cells into multiple organs, exhibiting significant clinical heterogeneity. Although standard chemotherapy regimens have markedly improved patient survival rates, several challenges remain, such as low response rates, high recurrence rates, and long-term sequelae in certain patients. This study aimed to integrate multimodal 18F-FDG PET/CT metabolic parameters, genetic markers, and clinical characteristics to evaluate and predict treatment efficacy and prognosis in patients with LCH.

**Methods:**

A retrospective analysis was conducted on clinical data and ^18^F-FDG PET/CT imaging findings from 26 patients diagnosed with LCH via biopsy pathology between May 2016 and December 2024 at the Department of Nuclear Medicine, Jiangxi Provincial People’s Hospital. Four metabolic parameters—SUVmax, TLR, MTV, and TLG—as well as genetic markers and clinical features (e.g., gender, age, type, stage) were evaluated. All patients were followed up for at least 1 year or until disease progression or relapse occurred. Univariate and multivariate analyses were performed to assess progression-free survival.

**Results:**

Patients with disease progression or recurrence exhibited significantly higher SUVmax, TLR, MTV, and TLG values compared to those who responded to treatment. ROC curve analysis identified optimal cutoff values for predicting disease remission as follows: SUVmax = 7.5, TLR = 5.2, MTV = 25.0, and TLG = 150. The remission rates in the high-value groups for SUVmax, MTV, and TLG were significantly lower than those in the corresponding low-value groups, with the most pronounced differences observed in the MTV and TLG groups (*p* < 0.01). TLG demonstrated the highest AUC value (0.91), indicating its strong predictive power. Clinicians should be vigilant about recurrence risk when MTV ≥ 25.0 or TLG ≥ 150.0. In univariate analysis, classification as multisystem LCH with risk-organ involvement (MS-LCH RO+), Ann Arbor stage III, BRAF V600E positivity, MTV > 25.0, and TLG > 150.0 were significant risk factors for worse progression-free survival (PFS) (all *p* < 0.05). Furthermore, patients in the high SUVmax, high MTV, and high TLG groups exhibited significantly shorter PFS. Multivariate Cox regression analysis identified the metabolic parameters MTV and TLG as independent predictors of PFS. The BRAF V600E mutation rate was significantly higher in patients with MS-LCH and those in the high SUVmax and high TLG groups.

**Conclusion:**

Baseline metabolic parameters derived from ^18^F-FDG PET/CT represent promising imaging biomarkers for predicting therapeutic response and prognosis in LCH. When integrated with established clinical stratification systems, these metabolic indices facilitate a more comprehensive multidimensional prognostic evaluation framework.

## 1 Introduction

Langerhans cell histiocytosis (LCH) is a rare clonal proliferative disorder characterized by the infiltration of pathological Langerhans cells into multiple organs, exhibiting significant clinical heterogeneity (1). Currently, chemotherapy remains the primary treatment modality for LCH, with high-risk patients requiring combination therapy involving glucocorticoids and targeted agents such as BRAF inhibitors (2). Although the standard chemotherapy regimen has markedly improved patient survival rates, certain challenges persist, including low response rates, high recurrence rates, and long-term sequelae in some patients (3). These variations in therapeutic efficacy are likely closely associated with underlying molecular genetic characteristics. Research has demonstrated that the BRAF V600E mutation occurs in approximately 50% of LCH cases, while abnormal activation of MAPK pathway-related genes (e.g., MAP2K1 and ARAF) has also been confirmed to contribute to disease progression and drug resistance (4). Consequently, elucidating the molecular mechanisms underlying these efficacy differences and establishing a precise evaluation system are critical for optimizing individualized treatment strategies.

Currently, the assessment of LCH treatment efficacy primarily relies on traditional imaging modalities (e.g., CT and MRI) combined with clinical indicators. However, these methods are limited to reflecting changes in anatomical structure and struggle to capture early-stage alterations in metabolic activity or small residual lesions (5). ^18^F-FDG PET/CT, as a functional imaging technique, enables noninvasive evaluation of disease activity by detecting glucose metabolism levels in lesions, offering unique advantages in monitoring therapeutic responses and predicting prognosis (6). Recent studies indicate that the integrated analysis of multi-modal metabolic parameters [e.g., SUVmax, metabolic tumor volume (MTV), and total lesion glycolysis (TLG)] may more comprehensively reflect tumor heterogeneity and dynamic therapeutic responses (7). Nevertheless, existing literature predominantly focuses on single-parameter analyses or short-term efficacy evaluations, lacking in-depth exploration of the correlations between comprehensive multi-parameter profiles and gene mutations.

In this study, we innovatively integrate ^18^F-FDG PET/CT multi-modal metabolic parameters with clinical and genetic mutation characteristics to comprehensively evaluate their roles in dynamic efficacy assessment and prognostic prediction of LCH, while exploring the potential mechanisms underlying efficacy disparities.

## 2 Data and methods

### 2.1 Clinical data

The clinical and ^18^F-FDG PET/CT imaging data from 26 patients diagnosed with LCH by biopsy pathology at our hospital between May 2016 and December 2024 were retrospectively analyzed. Among the 26 patients with LCH, there were 11 males and 15 females aged 0.5–66 years old, with a median age of 13 years old. And there were 12 adults and 14 children, including 11 children under the age of 6.

### 2.2 Imaging method

All patients fasted for more than 6 h, fasting blood glucose < 11.1 mmol/L, intravenous injection of ^18^F-FDG (dose 0.10–0.14 mCi/kg) in the wrist or elbow, and resting for 40–60 min, then ^18^F-FDG PET/CT examination was performed. CT scan (60-100 mA, 120 kV, 3.75 mm thickness) was performed first after bed. The scan range was from the top of the skull to the upper 1/3 of the femur, and the scan was performed to the sole if there were suspected lesions in the lower limbs. Then PET 3D model acquisition (6–8 beds, 3 min/bed), the range of the same as CT, image iteration reconstruction, and image attenuation correction based on CT; The images corrected by CT and PET were transferred to GE AW4.62 workstation for automatic fusion and related post-processing. The PET/CT model is GE Discovery STE. The second and subsequent ^18^F-FDG PET/CT examinations follow the same scanning conditions and methods. ^18^F-FDG was synthesized by the Nuclear medicine department of our hospital (GE MiniTrace cyclotron, Fastlab2 radiopharmaceutical automation synthesizer) with radiochemical purity > 95%.

### 2.3 Imaging analysis

The ^18^F-FDG PET/CT images were evaluated separately by two senior physicians or associate chief physicians of the nuclear medicine department. In case of disagreement, consensus was reached by consultation, or the final result was assessed by the higher-level physician.

#### 2.3.1 Qualitative analysis

The location, distribution, density, morphology of the lesions were observed on CT. The extent and distribution of FDG uptake were also studied. Higher uptake in the lesion compared to liver was considered abnormal.

#### 2.3.2 Quantitative analysis

The number of lesions was recorded on CT, and the size of lesions were measured, including the maximum diameter and the minimum diameter perpendicular to it. The maximum standard uptake value (SUVmax), SUVmean and Metabolic Tumor Volume (MTV), Total lession glycolysis (TLG) of lesions, liver and mediastinal blood pool was measured by PET. The 3D ROI margin threshold of tumor load was 40% × SUVmax. TLG = MTV × SUVmean. A SUVmax of the lesion greater than that of liver was considered abnormal. The portion of the hypermetabolic lesion on PET was outlined with a 10 -mm circle to delineate the Region of Interest (ROI). The liver SUVmean was measured and the Tumor-liver ratio (TLR) was calculated. TLR > 1 was considered as positive.

### 2.4 Treatment and follow-up

Patients underwent surgery or a systematic chemotherapy regimen, and some patients received targeted therapy for the BRAF V600E mutation (dalafenib). ^18^F-FDG PET/CT examination was performed within 1 month prior to treatment to determine staging and typing, and the first PET/CT examination was performed within 2 to 3 months after treatment to evaluate efficacy. Treatment response was evaluated according to international LCH study group criteria (8). Complete response (CR) means that the FDG uptake of the lesion is the same as that of the surrounding background tissue, or the abnormal imaging features are resolved; Partial response (PR) is when the lesion’s SUV is reduced from baseline, but continued uptake is higher than in the surrounding background tissue, or abnormal imaging features are reduced, but not completely disappeared; Disease progression/recurrence (DP/DR) is an increase in the value of the diseased SUV or a new FDG-positive lesion; Stable disease (SD) means that the other criteria above are not met. Progression-free survival (PFS) is defined as the time from initial diagnosis to first disease progression or recurrence. Patients were followed for at least 1 year until disease progression or recurrence.

### 2.5 Statistical analysis

All statistical processing was completed using SPSS23.0. Shapiro–Wilk test was used to test the normal distribution of measured data. Measurement data conforming to the normal distribution were expressed as x ± s, and independent sample t test was used for comparison between groups. Measurement data with non-normal distribution were represented by median (M) and quartile (P25, P75), and differences between groups were compared by Mann–Whitney U test or Chi-square test. ROC curve analysis was used to calculate the optimal threshold of each quantitative parameter with disease progression as the endpoint. Independent predictors of PFS were screened by Cox proportional hazard regression model. *p* < 0.05 was considered statistically significant.

## 3 Results

### 3.1 Clinical characteristics of patients

Among the 26 patients with LCH, the bone was the most involved site, up to 80.7% (21/26), followed by the lymph nodes (Table 1). There were 8 patients with SS-LCH and another 18 (69.2%) patients with MS-LCH, 7 of whom were accompanied by RO+. 65.4% of the patients carried the BRAF V600E mutation. Seven of the eight SS-LCH patients were treated with surgery, and the remaining 19 MS-LCH patients were all initially treated with systemic chemotherapy, with 10 BRAF V600E positive patients receiving targeted therapy at a later stage.

**Table 1:** Summary of clinical features of patients.

Features	Values (*n* = 26)
Age	
Adult	12
Children	14
<6 years old	11
Sex	
Male	11
Female	15
SS-LCH	8
MS-LCH	18
RO+	7
RO−	11
Infiltrating site	
Bones	21
Lymph nodes	9
Lung	4
Liver	5
Spleen	2
Pituitary gland	2
Skin	1
BRAF V600E mutation	17
Treatment method	
Surgery	7
Chemotherapy	19
Targeted therapy	10

### 3.2 Comparison of baseline PET metabolic parameters SUVmax, TLR, MTV and TLG between remission and progression/recurrence groups

Of the 26 patients, 6 progressed or relapsed (23.1%), 11 had complete response to CR (42.3%), and 9 had partial response to PR (34.6%) (Table 2). The SUVmax, TLR, MTV and TLG of patients with disease progression/recurrence were significantly higher than those in the response group (including complete and partial response), and the differences were statistically significant.

**Table 2:** Baseline PET metabolic parameters in the remission and progression/recurrence groups.

Metabolic parameters	Remission group	Progression/relapse group	*p* value
	(*n* = 20)	(*n* = 6)	
SUVmax	5.6 ± 3.1	8.7 ± 5.2	0.032^*^
TLR	4.1 ± 2.0	6.8 ± 3.6	0.015^*^
MTV (cm^3^)	17.3 ± 12.4	43.2 ± 35.7	0.008^**^
TLG (g)	96.5 ± 122.6	287.3 ± 210.5	0.006^**^

^*^p < 0.05, ^**^p < 0.01 (Mann–Whitney U test).

### 3.3 Predictive value of baseline metabolic parameters SUVmax, TLR, MTV, TLG and clinical features for therapeutic effect

ROC curve analysis (Table 3) showed that the best thresholds of SUVmax, TLR, MTV and TLG for predicting disease remission were 7.5, 5.2, 25.0 and 150, respectively. According to the optimal thresholds, patients were divided into high value group and low value group, in which the remission rate of the high value group of SUVmax, MTV and TLG was significantly lower than that of the corresponding low value group. The difference between the MTV and TLG groups was the most significant (*p* < 0.01). The AUC of TLG was the highest (0.91), and the risk of recurrence should be vigilant when MTV ≥ 25.0 or TLG ≥ 150.0. The remission rate was significantly higher in young patients (<18 years old), RO-, and stage I + II patients, and the difference was statistically significant (*p* < 0.05) (Table 4).

**Table 3:** ROC curve analysis and optimal cutoff value.

Metabolic parameters	AUC	Best cut-off value	Sensitivity	Specificity
SUVmax	0.82	≥7.5	83%	75%
TLR	0.78	≥5.2	80%	70%
MTV (cm^3^)	0.89	≥25.0	92%	85%
TLG (g)	0.91	≥150.0	95%	90%

**Table 4:** Clinical features and predictive value of BRAF V600E mutations for efficacy.

Variables	Grouping	Remission rate	X^2^	*p* value
Staging	I + II	64.3%	5.76	0.016
	III	90.0%		
Parting	MS-RO+	61.5%	6.89	0.009
	MS-RO−	90.9%		
BRAF mutations	Positive negative	68.4 80.0%	0.67	0.41
Gender	male female	70.0 76.5%	0.32	0.57
Age	Adult Children	58.3 85.7%	4.12	0.04

### 3.4 Univariate and multivariate analyses of baseline ^18^F-FDG PET/CT metabolic parameters predicting PFS

In univariate analysis, classification (MS-LCH RO+), stage (III), BRAF V600E positive, MTV > 25.0, TLG > 150.0 were significant risk factors for PFS (p < 0.05), and PFS was shorter in SUVmax, MTV, TLG high value group. COX multifactor regression analysis was performed for the statistically significant related factors in the univariate analysis, and the results showed that the metabolic parameters MTV and TLG were independent predictors of PFS (Table 5).

**Table 5:** Univariate and multivariate results of baseline 18F-FDG PET/CT metabolic parameters and clinical features in predicting PFS.

Features	Variables	Single factor analysis			Multifactor analysis		
		HR 95% CI		*p* value	HR 95% CI		*p* value
Clinical features	Gender (male/female)	1.12	0.45–2.78	0.81	1.05	0.39–2.81	0.92
	Age (≥18/<18 years old)	2.34	1.02–5.36	0.04	2.15	0.88–5.25	0.09
	Type (MS-LCH RO+/RO-)	3.67	1.52–8.85	0.003	3.02	1.21–7.55	0.02
	Stage (III/I + II)	4.21	1.74–10.2	0.001	3.58	1.42–9.03	0.007
Gene	BRAF (positive/negative)	2.89	1.21–6.89	0.02	2.45	1.01–5.97	0.048
Metabolic parameters	SUVmax (>7.5/≤7.5)	2.55	1.08–6.01	0.03	2.10	0.85–5.18	0.11
	TLR (>5.2/≤5.2)	1.98	0.83–4.72	0.12	1.65	0.67–4.06	0.27
	MTV (cm^3^) (>25.0/≤25.0)	3.10	1.28–7.49	0.01	2.75	1.10–6.87	0.03
	TLG (>150.0/≤150.0)	3.42	1.43–8.17	0.006	2.94	1.18–7.31	0.02

### 3.5 Clinical features, metabolic parameters and BRAF V600E gene positive versus negative cases

**Figure 1:**
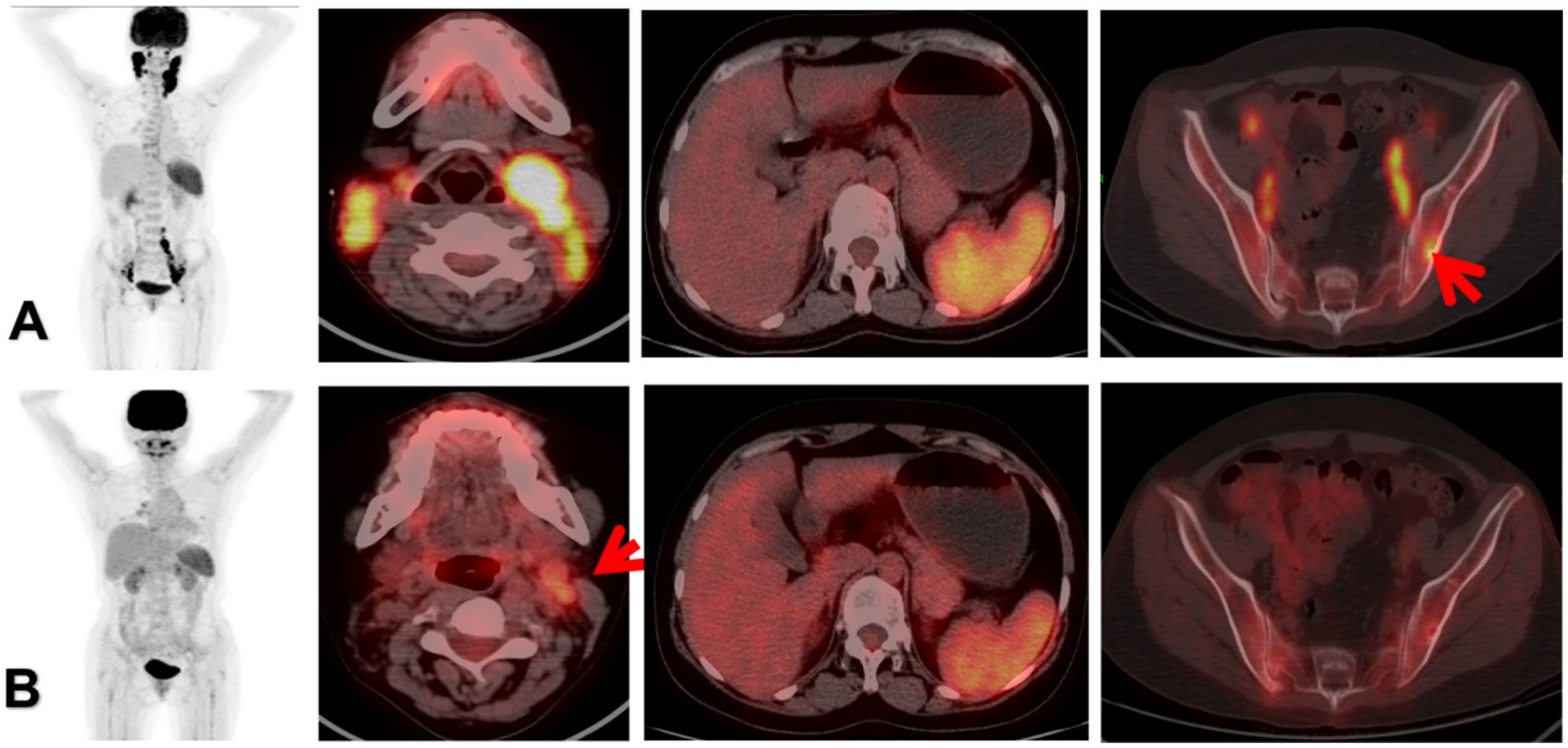
The patient was a 35-year-old female. Panel **(A)** was the 18F-FDG PET/CT before treatment, multiple enlarged lymph nodes were found in the bilateral neck, iliac vessels and retroperitoneum, with increased radioactive uptake. Among them, the SUVmax of the left cervical lymph nodes was 12.8, the MTV was 18.7, the TLR was 7.5, and the TLG was 152.3. Local bone destruction occurred in the left iliac bone, with increased metabolism, and the SUVmax was 4.8. The morphology and size of the spleen are normal, with increased diffuse radioactive uptake, and the SUVmax was 7.6. The clinical stage was stage III, classified as MS-LCH RO+, and BRAF V600E was positive. Panel **(B)** was reexamined after the treatment with the VP protocol. 18F-FDG PET/CT showed that the bilateral cervical lymph nodes were significantly reduced, the metabolism was decreased, and the metabolism of the spleen and the left iliac bone was significantly reduced, suggesting a significant therapeutic effect. However, a recurrence occurred during the follow-up visit half a year later. The red arrow of **(A)** indicated focal infiltration of the left ilium. The red arrow of **(B)** indicated that there was still mild FDG uptake in the left cervical lymph nodes, suggesting the presence of partial tumor activity.

BRAF V600E positive was more common in MS-LCH type and stage III patients (*p* < 0.01), and was significantly negatively correlated with SS-LCH type and early stage. The mutation rate of BRAF V600E gene was higher in MS-LCH, SUVmax and TLG high value groups, and there were statistical differences. The remission rate was 75% in BRAF V600E positive group and 83% in BRAF V600E negative group, and there was no statistically significant difference between the two groups (*p* = 1.000) (Table 6; Figure 1).

**Table 6:** Comparison of clinical features, metabolic parameters and BRAF V600E gene mutations.

Clinical features	Positive for BRAF V600E gene	Negative for BRAF V600E gene	X^2^	*p* value
Gender			0.52	0.47
Male	8 (47.1%)	3 (33.3%)		
Female	9 (52.9%)	6 (66.7%)		
Age of onset			1.2	0.27
>18 years old	9 (52.9%)	3 (33.3%)		
≤18 years old	8 (47.1%)	6 (66.7%)		
Involvements system			7.14	0.008
SS-LCH	2 (11.8%)	6 (66.7%)		
MS-LCH	15 (88.2%)	3 (33.3%)		
SUVmax			8.78	0.003
>7.5	5 (25%)	0		
≤7.5	15 (75%)	6 (100%)		
TLR			0.01	0.920
>5.2	5 (25%)	1 (16.7%)		
≤5.2	15 (25%)	5 (83.3%)		
MTV (cm^3^)			0.73	0.393
>25.0	3 (15%)	1 (16.7%)		
≤25.0	17 (85%)	5 (83.3%)		
TLG (g)			3.25	0.071
>150.0	4 (20%)	0		
≤150.0	16 (80%)	6 (100%)		

## 4 Discussion

This study systematically analyzed the clinical characteristics of 26 patients with Langerhans cell histiocytosis (LCH) and identified bone as the most frequently involved site (80.7%), aligning with findings from multiple recent studies (9, 10). Additionally, multisystem LCH (MS-LCH) constituted 69.2% of this cohort, with risk organ involvement (RO+) observed in 38.9% of cases, indicating a strong association between multisystem lesions and organ dysfunction. Heritier et al. (11) demonstrated a high prevalence of RO + among MS-LCH patients and a significantly elevated recurrence risk in this subgroup, consistent with the poorer prognosis observed in RO + MS-LCH patients in our cohort.

The BRAF V600E mutation detection rate in this cohort was 65.4% (17/26), marginally exceeding the previously reported 50% (12), potentially attributable to sample bias or methodological variations in mutation detection. Notably, 10 BRAF V600E-positive patients received targeted therapy (dabrafenib), whereas the BRAF-negative cohort primarily underwent chemotherapy, reflecting the influence of mutational status on therapeutic strategy. Diamond et al. confirmed that BRAF inhibitors significantly enhance progression-free survival (PFS) in mutation-positive patients, though efficacy diminishes when administered to RO+ patients (2). This observation correlates with our findings of comparable response rates between the BRAF-positive (75%) and BRAF-negative groups (83%, *p* = 1.00). This phenomenon could be explained by organ dysfunction in RO + patients negating the therapeutic advantages of targeted agents, compounded by limited statistical power due to the modest sample size.

This study demonstrated that baseline PET metabolic parameters (SUVmax, TLR, MTV, TLG) were significantly elevated in the progression/relapse group compared to the remission group among LCH patients (*p* < 0.05). Univariate and multivariate Cox regression analyses were employed to assess the predictive value of baseline 18F-FDG PET/CT metabolic parameters and clinical features for progression-free survival (PFS). Univariate analysis identified lesion type, disease stage, BRAF V600E positivity, and metabolic parameters MTV and TLG as significant risk factors for reduced PFS. Multivariate analysis further established MTV and TLG as independent prognostic predictors, exhibiting superior predictive power (HR = 2.75 and HR = 3.10, respectively) over BRAF V600E mutation (HR = 2.45, *p* = 0.048). These findings align with current evidence: a meta-analysis revealed MTV’s independent predictive value across multiple tumor types (pooled HR = 2.5, *p* = 0.02) (13), closely mirroring our MTV-specific results (HR = 2.75, *p* = 0.03).

The MTV threshold >25.0cm^3^ (HR = 2.75, p = 0.03) may indicate impaired drug penetration or clonal heterogeneity in bulky lesions–mechanisms extensively documented in lymphoma and solid tumors (14, 15). Notably, Kumar et al. (16) validated this threshold’s clinical relevance in lymphoma, associating MTV > 25 cm^3^ with elevated treatment failure risk. Similarly, TLG demonstrated robust prognostic stratification capacity, achieving an AUC of 0.91 in our study compared to Cottereau et al.’s reported AUC = 0.89 (17). Threshold analyses reinforced TLG’s predictive utility: Wang et al. identified baseline TLG > 150 as an independent progression predictor (HR = 4.2, *p* = 0.004) (18), while Park et al. established TLG > 200 as a significant PFS reduction marker (HR = 3.1, *p* = 0.01) across malignancies including LCH (19).

These collective results underscore the primacy of metabolic parameters over genetic markers in reflecting disease biology. Although BRAF V600E retained statistical significance in multivariate analysis (HR = 2.45, *p* = 0.048), its lower hazard ratio relative to MTV and TLG suggests metabolic indices more directly quantify tumor aggressiveness.

Although SUVmax and TLR demonstrated slightly lower predictive power compared to MTV and TLG (AUC 0.82 vs. 0.78, respectively), these parameters retained clinical utility for early therapeutic assessment. Notably, LCH patients with SUVmax > 7.5 exhibited significantly reduced remission rates (*p* = 0.032), corroborating Park et al.’s findings that SUVmax reflects tumor proliferative activity and potential BRAF mutation associations (19). However, our study failed to establish a statistically significant correlation between BRAF V600E mutation and TLR. TLR’s dependency on hepatic metabolic stability introduces confounding factors unrelated to tumor genomics. In contrast, SUVmax and TLG directly quantify tumor-intrinsic glycolytic activity, aligning with BRAF-driven metabolic reprogramming.

Clinical feature analysis revealed significantly higher response rates in younger patients (<18 years), early-stage (stage I-II) cases, and MS-LCH RO− subtypes (all *p* < 0.05). The superior response rate in pediatric patients (85.7% vs. 58.3% in adults) may reflect age-related advantages in immune function and chemotherapy sensitivity, aligning with Allen et al.’s mechanistic insights (20). Conversely, MS-LCH RO + patients showed markedly lower response rates (61.5% vs. 90.9% in RO− group), underscoring risk organ involvement as an independent adverse prognostic factor necessitating intensified monitoring. Intriguingly, BRAF V600E mutation status showed no significant association with treatment response (*p* = 0.41), despite 52.9% of mutation-positive patients having MS-LCH RO + and receiving targeted therapy. This observation aligns with Diamond et al.’s (2) report that BRAF inhibitor efficacy diminishes in RO+ patients or those with resistant mutations. Prognostic analysis confirmed significantly shortened PFS in MS-LCH RO + (HR = 3.02, *p* = 0.02) and stage III patients (HR = 3.58, *p* = 0.007), consistent with Morimoto et al. and Donadieu et al.’s (3, 21) consensus regarding risk organ involvement and multisystem disease as pivotal prognostic determinants.

Comparative analysis of BRAF V600E mutation-positive and negative cohorts revealed distinct clinicometabolic profiles in LCH patients. The mutation-positive group exhibited significantly higher prevalence of multisystem LCH (88.2% vs. 33.3%, *p* = 0.008) and advanced-stage disease (*p* < 0.01), aligning with Nelson et al.’s findings of elevated BRAF mutation rates in MS-LCH versus single-system LCH (60% vs. 30%) (4). Diamond et al. (2) further established that BRAF mutations drive MAPK pathway hyperactivation, potentially facilitating multisystem invasion and disease progression.

Notably, no significant difference in treatment response was observed between mutation-positive (75%) and negative groups (83%, *p* = 1.000). This paradoxical finding may be explained by: (1) High-risk organ involvement (RO+) in 52.9% of mutation-positive patients potentially neutralizing targeted therapy benefits; (2) Development of resistance mechanisms through secondary MAPK pathway mutations or epigenetic dysregulation, as documented in recent studies (22). Emerging evidence suggests BRAF mutation status requires integration with tumor microenvironment biomarkers (e.g. PD-L1 expression) for precise therapeutic prediction (23).

Metabolic profiling demonstrated significantly higher SUVmax >7.5 prevalence in mutation-positive patients (25% vs. 0%, *p* = 0.003), with a trend toward elevated TLG > 150.0 frequency (20% vs. 0%, *p* = 0.071). These observations corroborate Park et al.’s (24) findings of enhanced SUVmax in BRAF-mutated LCH (8.2 vs. 5.1, *p* = 0.02), potentially reflecting MAPK-mediated metabolic reprogramming. Mechanistically, *in vitro* studies confirm BRAF V600E mutations upregulate glycolytic enzyme expression, augmenting FDG avidity (25), thereby providing molecular substantiation for our metabolic findings. The number of BRAF V600E negative cases in this article was relatively small. The possible reason was that there were more adult patients, the cohort dominated by pediatrics inherently showed a higher positive BRAF V600E (26), Spatiotemporal heterogeneity in LCH lesions might lead to false-negative biopsies, BRAF-negative cases might harbor alternative MAPK-pathway mutations (e.g., MAP2K1, ARAF), obscuring the true driver biology (27).

This study acknowledges several limitations inherent in its design. As a single-center retrospective cohort study with limited enrollment (*n* = 26), particularly noting the small BRAF-negative subgroup (*n* = 9), the generalizability of findings requires validation through multi-institutional trials. Furthermore, constrained follow-up duration and the absence of advanced PET biomarkers (e.g., radiomic features) or comprehensive molecular profiling (including non-canonical MAPK pathway alterations) restricted longitudinal survival analysis and late-effect assessment. Future mechanistic investigations should incorporate longitudinal multi-omics integration to elucidate dynamic tumor-host interactions.

## 5 Conclusion

Our findings establish multimodal PET metabolic parameters, particularly TLG and MTV, as useful predictors of therapeutic response and progression-free survival in LCH. When integrated with clinical stratification systems, these indices form a multidimensional prognostic evaluation framework. While BRAF V600E mutation status showed no direct therapeutic correlation, its predominant association with multisystem involvement underscores the pathogenic significance of MAPK pathway dysregulation. The synergistic interpretation of functional imaging signatures, molecular profiling, and clinical parameters provides a scientific foundation for developing precision stratification protocols in LCH management. However, more research is still needed for further verification.

## Data Availability

The datasets presented in this study can be found in online repositories. The names of the repository/repositories and accession number(s) can be found in the article/supplementary material.
